# Mutations in the E2-PePHD region of hepatitis C virus genotype-3a and correlation with response to interferon and ribavirin combination therapy in Pakistani patients

**DOI:** 10.1186/1743-422X-7-377

**Published:** 2010-12-31

**Authors:** Samia Afzal, Muhammad Idrees, Madiha Akram, Zunaira Awan, Bushra Khubaib, Mahwish Aftab, Zareen Fatima, Sadaf Badar, Abrar Hussain

**Affiliations:** 1 National Centre of Excellence in Molecular Biology, 87-West Canal Bank Road Thokar Niaz Baig Lahore-53700, University of the Punjab, Lahore, Pakistan

## Abstract

Hepatitis C is a major health problem affecting more than 200 million individuals in the world. Current treatment regimen consisting of interferon alpha and ribavirin does not always succeed in eliminating the virus completely from patient's body. One of the mechanisms by which virus evades the antiviral effect of interferon alpha involves protein kinase (PKR) eukaryotic initiation factor 2 alpha (eIF2a) phosphorylation homology domain (PePHD). This domain in genotype 1 strains is reportedly homologous to PKR and its target eIF2a. By binding to PKR, PePHD inhibits its activity and therefore cause virus to evade antiviral activity of interferon (IFN). Many studies have correlated substitutions in this domain to the treatment response and lead to inconclusive results. Some studies suggested that substitutions favor response while others emphasized that no correlation exists. In the present study we therefore compared sequences of PePHD domain of thirty one variants of six hepatitis C virus patients of genotype 3. Three of our HCV 3a infected patients showed rapid virological response to interferon alpha and ribavirin combination therapy whereas the remaining three had breakthrough to the same combination therapy. It is found that PePHD domain is not entirely conserved and has substitutions in some isolates irrespective of the treatment response. However substitution of glutamine (Q) with Leucine (L) in one of the breakthrough responders made it more identical to HCV genotype 1a. These substitutions in the breakthrough responders also tended to increase average hydrophilic activity thus making binding of PePHD to PKR and inhibition of PKR more favorable.

## Findings

Hepatitis C Virus (HCV) is a major health concern worldwide with current estimates of more than 200 million affected individuals [1]. In Pakistan 17 million people are infected and about 20% are carriers for HCV [2]. In 60-80% cases HCV may lead to hepatocellular carcinoma (HCC) [3]. It comprises of 9600 nucleotides that predetermines a polypeptide containing 3010-3033 amino acids and encode 3 structural (Core, E1, E2) and 7 nonstructural (p7, NS2, NS3, NS4A-B, and NS5A-B) proteins [4].

Current approved therapy for HCV is interferon alpha [5] in combination with ribavirin [6] administered for 24 to 48 weeks but it does not eliminate virus completely in 50-80% of the patients [7]. Many viral and host factors are involved in the response to interferon therapy. Viral factors that favor sustained virological response to IFN therapy includes HCV genotypes other than genotype 1 and low viral load [8].

After death of cells interferon is released and in response neighboring cells release PKR [9]. One of the mechanisms by which IFN hamper HCV replication involves protein kinase (PKR) which is activated by double stranded RNA. Interferon alpha induces autophosphorylation of protein kinase by binding to it and phosphorylated PKR which in turn phosphorylates eukaryotic initiation factor 2 alpha (eIF2a) and as a consequence HCV RNA transcription is halted. However HCV has also evolved certain mechanisms to overcome the antiviral activity of interferon. Such as E2 protein that carries a 12 amino acid domain called as PKR-eIF2a phosphorylation homology domain (PePHD) which binds to PKR and inhibits its activity thereby inhibiting the antiviral effects of interferon alpha which ultimately leads to viral persistence. This binding is because of the similarity of this PePHD domain with phosphorylation domain of PKR and eIF2a [10].

In order to find out whether PePHD region of E2 gene shows any promising results for interferon treatment response of HCV patients we investigated six HCV patients. Baseline serum samples from six HCV patients of genotype 3a subjected to Interferon alpha and ribavirin combination therapy. Three patients (R1, R2, and R3 were rapid responders characterized by negative HCV RNA (>500 IU/ml) after 4 weeks of treatment. Two patients (BT) were breakthrough virological responders characterized by reappearance of HCV RNA at the end of treatment. One of the patients was defined as end of treatment responder (ETR) as characterized by negative HCV RNA at the end of treatment. E2 gene amplified from all these samples was cloned. Five to nine variants from each sample were sequenced and analyzed. Purified PCR product was sequenced by using ABI prism sequencer. Consensus sequences were generated using BioEdit software. PePHD amino acid sequences of all variants were aligned with multiple alignment tools using CLC workbench software http://www.clcbio.com. Amino acid composition was calculated using MEGA version 4.1.

**Figure 1:**
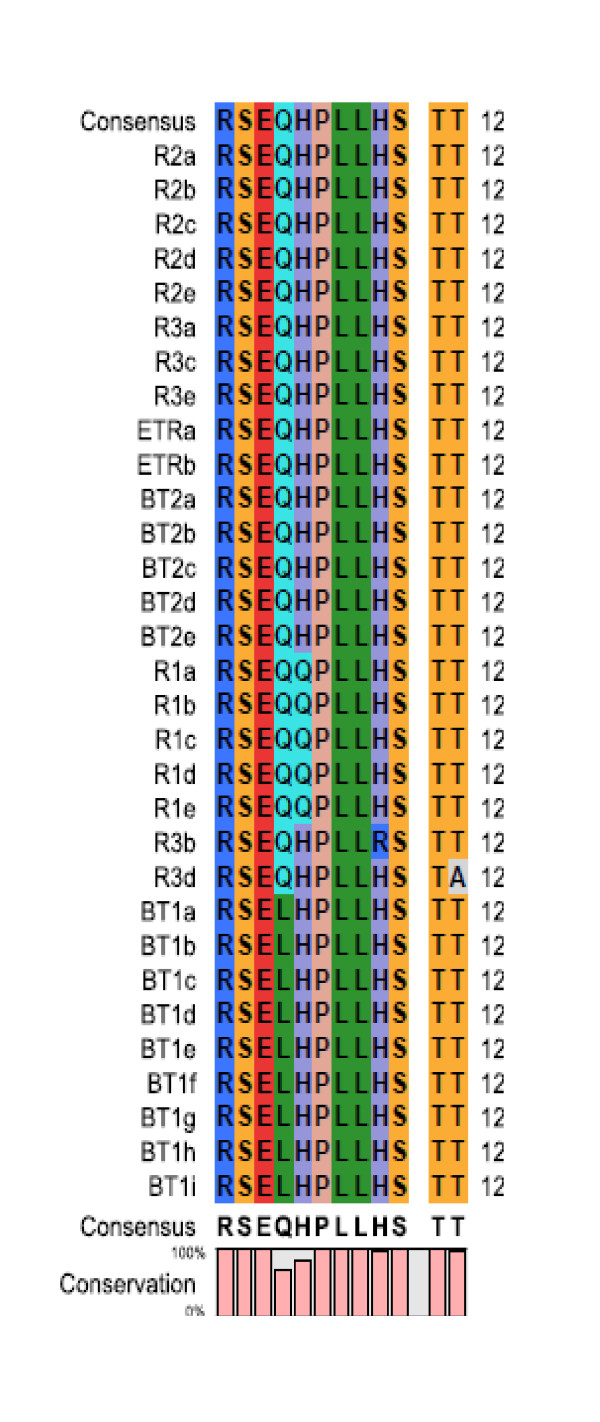
**PePHD amino acids multiple alignment of 31 variants of six HCV genotype 3a baseline samples subjected to IFN alpha and Ribavirin combination therapy**.

The subject is very controversial as some of the investigators reported a correlation between amino acid substitution in PePHD domain of HCV genotype 1, 2 and 3 strains [11-13] and treatment responses whereas its has been shown in some others found that PePHD domain is a conserved domain and no correlation exists between amino acid substitution and treatment response [14-17]. In this study we aligned amino acid sequences of PePHD domain from thirty one variants of six HCV genotype 3 strains including consensus sequences (Figure. 1). The region was found to be conserved with amino acid substitution at only two amino acid positions 4 and 5. At position 4 glutamine (Q) was replaced by Leucine (L) in variants of one of the breakthrough (BT1) sample (Table 1) and at position 5 histidine (H) got replaced with Q in variants of one of the rapid responder (R1) (Tables 2). Other amino acid positions were almost conserved with either no substitution or very rare substitution in any one of the variants. Since substitutions were found in both rapid responders and breakthrough responders, therefore this finding is consistent with earlier reported substitutions in PePHD which are not correlated to treatment response [14-17]. Amino acid sequences of PePHD domain in HCV genotype 1a and 1b are shown in table 3 to which the mutations were compared.

**Table 1:** PePHD amino acid substitutions in base line samples of break through responders to interferon plus ribavirin therapy (14 variants)

consensus	R	S	E	Q	H	P	L	L	H	S	T	T
	R14	S14	E14	L9	H14	P14	L14	L14	H14	S14	T14	T14

				Q5								

**Table 2:** PePHD amino acid substitutions in base line samples of rapid responders to interferon plus ribavirin therapy (15 variants)

consensus	R	S	E	Q	H	P	L	L	H	S	T	T
	R15	S15	E15	Q15	H10	P15	L15	L15	H14	S15	T15	T14

					Q5				R1			A1

**Table 3:** Amino acid sequences of PePHD domain in HCV genotype 1a and 1b.

HCV j strain 1b	R	S	E	L	S	P	L	L	L	S	T	T
HCV 1a	R	S	E	L	S	P	L	L	L	T	T	T

Taylor et al reported in vitro inhibition of PKR due to similarity of genotype 1 PePHD domain and phosphorylation domain of PKR and eIF2a [18]. In our local HCV isolates of 3a genotype, PePHD domain in those responding rapidly to treatment and those showing a breakthrough response were compared with PePHD domain of HCV 1a strain. Genotype 3a PePHD carries amino acids glutamine at position 4 and histidine at position 5 and 9. These three amino acid positions are important since amino acid substitutions are common at these positions. In our one breakthrough responder, glutamine is replaced by leucine making it more identical to PePHD domain of HCV genotype 1 strain which could be possible reason of HCV persistence in these patients. However no such substitution was seen in other strain with the same response. Therefore we can predict that apart from PePHD binding to PKR and inhibiting antiviral activity of IFN alpha, other factors might also be involved in establishing the response rates to anti-viral treatment. Additional investigations should be carried out for through comprehension of the study of these unknown factors and mechanisms involved in treatment response.

Average amino acid composition of polar, non polar and neutral amino acids was compared between samples responding differently to the treatment (Table 4). Comparison between breakthrough and rapid responder group of patients indicated that composition of polar amino acids in rapid responders (74.44%) was higher than breakthrough responders (69.65%). On the whole polar amino acids were greater in composition than non polar amino acids in all samples. Among polar amino acids positively charged amino acids were greater than negatively charged amino acid thus making it a basic stretch that might be involved in interacting with some negatively charged proteins. Polar basic amino acid composition was slightly higher in breakthrough responders (25.00%) than in rapid responders (22.22%) and that was due to the substitution of basic amino acid histidine with a polar neutral amino acid glutamine in one of the rapid responders. So this substitution ultimately leads to change in the average amino acid composition of PePHD. The same substitution also led to change in the composition of hydrophilic amino acids between rapid responders and non responders (Table 5) Average composition of hydrophilic amino acid was higher in breakthrough responders (33.33%) than rapid responders (30.55%). In one of the study conducted on HCV 3a genotype strain substitutions were found in the hydrophilic area (codon 668 and 669), where hydrophilic amino acids were replaced by hydrophobic amino acids in sustained responders [11]. In our local 3a strains, substitutions in rapid responder were found in hydrophilic amino acid histidine which was replaced by a neutral amino acid glutamine. Another amino acid substitution observed in breakthrough responders at position 4, a polar neutral amino acid glutamine being replaced by non polar hydrophobic amino acid leucine. But still both the substitutions can be significant as far as hydrophilicity of PePHD is concerned. Both substitutions resulted in replacement of hydrophilic and basic amino acids with a neutral amino acid in rapid responders and replacement of a neutral amino acid with hydrophilic and basic amino acid in breakthrough responders. Consequently both substitutions manifested an inverse relationship for the hydrophilic character based upon amino acid composition i.e. increased in breakthrough responders and decreased in rapid responders. This changed hydrophilicity may affect the potential interactions both in breakthrough and rapid responders.

**Table 4:** Amino acid composition of non-polar, neutral and polar basic and acidic amino acids in rapid responders (15 variants) and in breakthrough responders (BT)

PePHD	Polar a.a composition			Non polar a.a composition
	
	+ve charged (basic)	- ve charged (acidic)	Neutral	
RR group (15 variants)	Lys(K) 0.00	Asp(D) 0.00	Ser(S) 16.67	Ala(A) 0.56
	
	His(H) 13.33	Glu(E) 8.33	Thr(T) 16.11	Val(V) 0.00
	
	Arg(R) 8.89		Gln(Q) 11.11	Leu(L) 16.67
	
			Cys(C) 0.00	Ile(I) 0.00
	
			Asn(N) 0.00	Gly(G) 0.00
	
			Tyr(Y) 0.00	Trp(W) 0.00
	
				Phe(F) 0.00
	
				Pro(P) 8.33
	
				Met(M) 0.00

Average composition	22.22	8.33	43.89	25.56

BT group	Lys(K) 0.00	Asp(D) 0.00	Ser(S) 16.67	Ala(A) 0.00
	
	His(H) 16.67	Glu(E) 8.33	Thr(T) 16.67	Val(V) 0.00
	
	Arg(R) 8.33		Gln(Q) 2.98	Leu(L) 22.02
	
			Cys(C) 0.00	Ile(I) 0.00
	
			Asn(N) 0.00	Gly(G) 0.00
	
			Tyr(Y) 0.00	Trp(W) 0.00
	
				Phe(F) 0.00
	
				Pro(P) 8.33
	
				Met(M) 0.00

Average composition	25.00	8.33	36.32	30.35

**Table 5:** Amino acid composition of hydrophilic and hydrophobic amino acids in rapid responders (15 variants) and in breakthrough responders (BT)

	Hydropathic composition		
	
PePHD	Hydrophobic	Neutral	Hydrophillic
RR group (15 variants)	Leu (L) 16.67	Thr (T) 16.11	Arg (R) 8.89
	
	Ile (I) 0.00	Glu (E) 8.33	Lys (K) 0.00
	
	Phe (F) 0.00	Gly (G) 0.00	Asn (N) 0.00
	
	Trp (W) 0.00	Ser (S) 16.67	His (H) 13.33
	
	Val (V) 0.00	Gln (Q) 11.11	Pro (P) 8.33
	
	Met (M) 0.00	Asp (D) 0.00	
	
	Cys (C) 0.00		
	
	Tyr (Y) 0.00		
	
	Ala (A) 0.56		

Average composition	17.23	52.22	30.55

BT group	Leu (L) 22.02	Thr (T) 16.67	Arg (R) 8.33
	
	Ile (I) 0.00	Glu (E) 8.33	Lys (K) 0.00
	
	Phe (F) 0.00	Gly (G) 0.00	Asn (N) 0.00
	
	Trp (W) 0.00	Ser (S) 16.67	His (H) 16.67
	
	Val (V) 0.00	Gln (Q) 2.98	Pro (P) 8.33
	
	Met (M) 0.00	Asp (D) 0.00	
	
	Cys (C) 0.00		
	
	Tyr (Y) 0.00		
	
	Ala (A) 0.00		

Average composition	22.02	44.65	33.33

## Conclusion

We conclude that PePHD domain in our local HCV 3a strains is not totally conserved; it carries substitutions in some samples irrespective of their response to alpha interferon. However substitutions are such that it tend to decrease the average hydrophilic activity of PePHD domain in rapid responders and increase the average hydrophilic activity in breakthrough responders. Additionally the comparative greater similarity of PePHD domain in breakthrough responders with PePHD domain of genotype 1 made it a more efficient candidate for binding to and inhibiting PKR. This leads HCV to persist by evading antiviral activity of interferon alpha.

## Competing interests

The authors declare that they have no competing interests.

## Authors' contributions

SA and MI conceived of the study participated in its design and coordination and gave a critical view of manuscript writing. SA collected samples, epidemiological data, perform all molecular biology assays and analyzed the data statistically. MA,ZA, BK, MA, ZF, SB and AH participated in data analysis. All the authors read and approved the final manuscript.
